# Capacity Model and Constraints Analysis for Integrated Remote Wireless Sensor and Satellite Network in Emergency Scenarios

**DOI:** 10.3390/s151129036

**Published:** 2015-11-17

**Authors:** Wei Zhang, Gengxin Zhang, Feihong Dong, Zhidong Xie, Dongming Bian

**Affiliations:** College of Communication Engineering, PLA University of Science and Technology, 88 Houbiaoying Rd. Nanjing 210007, China; E-Mails: zev@msn.com (W.Z.); dfh_sinlab@hotmail.com (F.D.); xzd313@163.com (Z.X.); bian_dm@163.com (D.B.)

**Keywords:** capacity, remote wireless sensor networks, satellite network, emergency communications, integrated remote wireless sensor and satellite network

## Abstract

This article investigates the capacity problem of an integrated remote wireless sensor and satellite network (IWSSN) in emergency scenarios. We formulate a general model to evaluate the remote sensor and satellite network capacity. Compared to most existing works for ground networks, the proposed model is time varying and space oriented. To capture the characteristics of a practical network, we sift through major capacity-impacting constraints and analyze the influence of these constraints. Specifically, we combine the geometric satellite orbit model and satellite tool kit (STK) engineering software to quantify the trends of the capacity constraints. Our objective in analyzing these trends is to provide insights and design guidelines for optimizing the integrated remote wireless sensor and satellite network schedules. Simulation results validate the theoretical analysis of capacity trends and show the optimization opportunities of the IWSSN.

## 1. Introduction

Emergency scenarios can benefit from the deployment of a remote wireless sensor network (WSN) in the target area for a two-fold task: (1) gathering important information from the field; and (2) supporting audio, video and data communication when other terrestrial systems are not available. Additionally, sensor devices are frequently complemented by additional multimedia traffic sources (*i.e*., laptop computers, cameras and smart phones) [1,2]. Emergency communications can provide information transfer services for rescuers and victims in disasters using various sensor devices through a remote WSN [3,4]. The capability to transmit massive amounts of information (e.g., video, voice and data) back is essential to improve the coordination of rescuers during an emergency crisis and the response efforts.

A satellite network can operate independently from terrestrial infrastructure. When terrestrial outages occur from man-made and natural events, satellite connections remain operational. Satellite networks have a number of potential advantages over conventional technologies [5,6], including: (1) global availability; (2) high reliability; (3) immediacy; and (4) scalability. Hence, satellites are vastly underused and can be utilized for various uses to construct an integrated remote wireless sensor and satellite network in extreme conditions [7,8]. Integrated remote wireless sensor and satellite networks are expected to optimally meet the emergency information requirements of emergency relief and recovery operations for tackling large-scale disasters [9]. Due to the distinguishing characteristics of integrated remote wireless sensor and satellite networks, how to make these satellites and remote wireless sensors cooperate efficiently is challenging and important. We study this problem by evaluating the integrated sensor and satellite network capacity and find opportunities to optimize the network schedules from the capacity trends.

Since the seminal work of Gupta and Kumar [10], extensive research has been done in network capacity. However, most existing works are about ground networks, and there are limited works exploring the potential for sensor and satellite network capacity. Sara *et al.* [11] studied the models and tools to assess the communication capacity for geographically-diverse ground stations that loosely collaborate. In particular, they considered a specific ground-to-space scenario and optimized the ground station network. Nishiyama *et al.* [12] proposed a distributed traffic load strategy based on network capacity estimation for a multi-layered satellite network. They also assumed a specific scenario where the inter-satellite links (ISLs) are lattice connected. Liu *et al.* [13] proposed a mathematical framework to formulate the relationship between the network capacity and architectural parameters for a two-layered low earth orbit (LEO) and middle earth orbit (MEO) satellite network. Chen *et al.* [14] analyzed the satellite communication network characteristics and pointed out that the characteristics will affect the network capacity. Furthermore, there are other works studying the satellite or ground node network capacity [15,16], but they lack universal properties that can be extended to study the capacity of dynamic heterogeneous integrated remote wireless sensor and satellite networks. We need a general, analytical model that enables us to explore the impact of dynamic parameters on remote sensor and satellite network capacity.

In this article, we study the capacity problem of an integrated remote wireless sensor and satellite network (IWSSN). We propose a general capacity definition and analytic model for the IWSSN. Our model provides an accurate and efficient method to understand the effect of the network dynamics, e.g., the orbital dynamics of the satellite or new satellite injections into existing constellations. We sift through major capacity-impacting constraints and analyze the influence of these constraints. With this model, we can rigorously study the time-varying network capacity trends and limitations of the remote sensor and satellite network. We combine the geometric satellite orbit model and satellite tool kit (STK) engineering software to study the major factors influencing network capacity on short-term and long-term potential. Our objective in analyzing the capacity trends is to provide insights and design guidelines for optimizing the IWSSN network schedules.

The rest of this article is organized as follows. Section 2 summarizes related works in integrated sensor-satellite networks and existing capacity models/tools. Section 3 gives a formal definition of the network model and notations. In Section 4, we propose a general capacity definition and an analytical capacity model for the IWSSN. Then, we analyze the influence of major capacity-impacting constraints on the network capacity in Section 5. Finally, we make a conclusion in Section 6.

## 2. Related Works

### 2.1. Integrated Sensor and Satellite Network

Recently, wireless sensor networking has emerged as a low-cost technology for unmanned monitoring of a wide range of environments [17]. These sensor nodes usually consist of sensing, data processing and communication components, which can monitor and process physical data, such as temperature, humidity, vibrations, sounds, pictures and other data [18,19]. However, these low-cost sensors usually have a short ground transmitting range. They transmit information to respective base stations. Then,these base stations send the data to the receiver over a terrestrial network [20]. In areas that lack the appropriate terrestrial infrastructure [21], such as a disaster area, desert areas, ocean areas, *etc*., it is hard for sensors to transmit their information to the destination over a terrestrial network. Satellite links are an essential element of long-distance telecommunications [22]. Satellites have been used to provide many services, such as mobile communications, television, broadband Internet services, *etc*. It is envisaged that in the future, satellite networks will be integrated with terrestrial ones in order to provide a wider coverage area and data transmission to or from remote, inaccessible areas [23].

Integration of existing terrestrial sensor networks and satellite networks is one kind of integrated terrestrial and satellite network. It allows senors achieving ubiquitous information exchange between geographically-separated sites at an affordable cost. How to design and optimize the integrated sensor and satellite network schedules provides the first step in order to have a seamless and efficient integration between different technologies. In [24,25,26], Bisio *et al.* studied the information distribution methods in integrated sensor and satellite networks. They developed methods to select the Earth station that assures the improvement of network performance in terms of energy consumption, load and total time spent by information packets in the network. In [27], Yuan *et al.* proposed a delay tolerant network (DTN) bundle layer implementation for the throughput enhancement in integrated sensor and satellite networks. They used the bundle layer to solve the problem of data transmission efficiently. In [28], Verma *et al.* studied the performance of different integrated sensor and satellite network architectures. The packet loss rate and average end-to-end packet delay were compared in different network architectures. In [29], Vassaki *et al.* considered the integration of a dense machine to machine (M2M) sensor network and satellite network. The M2M sensor devices are locally grouped into clusters and communicate with satellites. Although integrated terrestrial and satellite networks have been studied in many works, to our best knowledge, there are no previous results on how to evaluate the integrated sensor and satellite network capacity.

### 2.2. Existing Capacity Models and Tools

As described in the Introduction section, extensive research has been done in network capacity. Cai *et al.* [30] focused on the ultra-wideband-based wireless personal area network capacity using a three-dimensional model. Wang *et al.* [31] solved the problem of under which scenarios a large network capacity gain can be expected by using higher transmission power. Urgaonkar *et al.* [32] investigated the network capacity region for a delay-tolerant mobile *ad hoc* network. Mao *et al.* [33] proposed a general capacity model for both static and mobile wireless networks. Their results are valid for both finite networks and asymptotically infinite networks. Aguirre *et al.* [34] considered the deployment strategy problem for wireless sensor networks to get a large network capacity. Jiang *et al.* [35] studied the the optimal source selection strategy to enhance the network capacity, where an optimization model is proposed to find the optimal source selection probability distribution. However, the surveyed existing literature discusses network capacity models, and prior works mainly focus on ground networks. Limited or no works have been done to develop a general analytical model for the capacity of dynamic, heterogeneous integrated sensor and satellite networks.

Current software tools provide calculation abilities for modeling and simulating aspects of spacecraft and ground node systems. However, no integrated tool exists for network capacity study. That is, tools do not use analytical models for assessing network capacity and are unable to simulate the capacity of dynamic networks. For example, the satellite tool kit (STK) developed by Analytical Graphics Incorporated (AGI) can calculate high fidelity spacecraft and ground node information, including position, velocity, visibility, revisit times, *etc*. However, STK itself cannot calculate capacity numbers directly. In reference [36], Beering *et al.* discussed the database structure of the communications system taxonomy (CommTax) toolkit, which uses STK and the QualNet network tool to model communication links among network nodes. In reference [37], Matar studied the channel capacity problem in LEO remote sensing satellite systems. They improved the channel capacity of the system on the basis of STK. In our work, we will combine STK and MATLAB to quantify the trends of the capacity constraints in our capacity model. In Section 5, we will discuss this in detail.

## 3. Network Model

**Figure 1:**
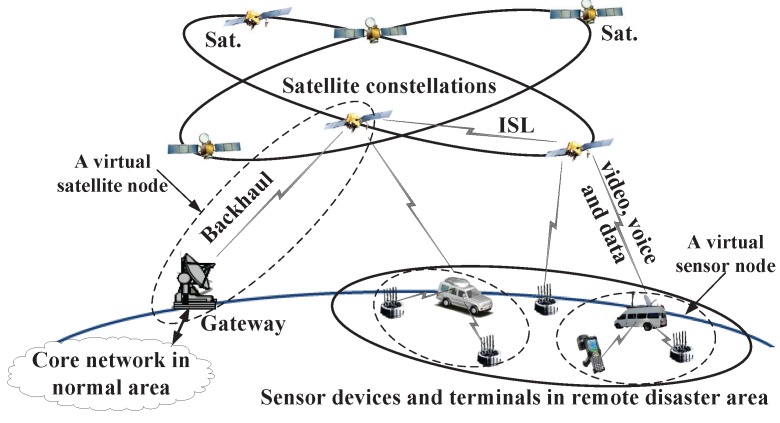
The architecture of an integrated remote wireless sensor and satellite network for emergency scenarios.

The architecture and network model of the IWSSN for emergency communications is depicted in this section. When a disaster occurs, an emergency information system will be established, as shown in Figure 1, which mainly consists of three parts, *i.e*., satellite constellations, sensor devices and a core network in a normal area. Sensor devices and terminals in the remote disaster area transmit large amounts of information (e.g., video, voice and data) back to the core network in the normal area using satellites as relays. Satellites can exchange information with each other utilizing inter-satellite links (ISLs).

In order to get a general analytic network model, we use the term arbitrary sensor and satellite network to refer to such a remote sensor and satellite network with a total of *N* nodes arbitrarily and deterministically (*i.e*., not randomly) located in orbits (LEO, MEO, geostationary earth orbit (GEO), *etc*.) or distributed in remote disaster area (sensor nodes). Obviously, these nodes may be either stationary (sensor nodes) or in motion following arbitrary and fixed (*i.e*., not random) trajectories (satellite nodes), so we use the term arbitrary. Each node in the network can be considered as a source, a relay, a destination or a mixture. A node may choose an arbitrary and fixed number of other nodes as its destinations. Considering the case that a source has multiple destination nodes, the source node may transmit the same information to its destination nodes, *i.e*., multicast, or transmit different information to different destination nodes, *i.e*., unicast. In emergency scenarios, sensors and satellites in the network generally have the ability to collect great amounts of data. It is assumed that all of the nodes are capable of sourcing and sinking infinite amounts of data, *i.e*., there is always information waiting at the source nodes, and received information can always be processed. This saturated traffic scenario enables us to isolate unique characteristics of sensor and satellite nodes that influence network capacity.

Let $V_{N}$ be the node set, and let *E* be the link set. A link in the arbitrary sensor and satellite network is a means of an information channel connecting one node to another for the purpose of exchanging mission-specific data (video, voice, data, *etc*.). The establishment of a link may follow either the protocol model or the physical model. As we assess sensor and satellite network capacity, we do not consider links between sensors for three reasons. (1) sensor nodes connected to each other can be taken as one virtual source or destination node, as shown in the following Figure 1; also, the gateway and the satellite can be taken as one virtual source or destination node; (2) links between sensor nodes usually exist, but information in a disaster area cannot go back to the the core network in the normal area without the help of satellites in most emergency scenarios; (3) the analysis of the network capacity between sensors helps us little to provide guidelines for optimizing the IWSSN schedules. Additionally, it will make the network capacity model complicated. Hence, we only consider links between sensor and satellite, satellite and satellite. Additionally, we are interested in the overall ability of the network to move data (bits) and are not concerned with the type of data (control or service). Links between sensor and satellite usually are a Rician fading channel. Additionally, links between satellite and satellite are an AWGN channel. Hence, we assume a line-of-sight must exist between two nodes when the link exists (Links may be feasible without direct line-of-sights, e.g., the satellite telephone service of the Iridium system [38,39]; however, in most practical space scenarios, line-of-sight is necessary; the special cases are not considered in our current work). The existence and bandwidth of the links between sensor and satellite nodes are time variant, related to the dynamics and constraints of the satellite orbit. The link set *E* at a particular time instant *t* may be more appropriately denoted by $E\left(t\right)$ to emphasize the temporal dependence. In this article, we drop $\left(t\right)$ for convenience.

Without loss of generality, we further assume that each node *n*, where n∈N, can transmit at a maximum data rate $R_{n}\left(t\right)$ bit/s at time *t* over a common information channel. It is immaterial to the capacity result if the channel is broken into several sub-channels of capacity $R_{n}^{\left(1\right)}\left(t\right),R_{n}^{\left(2\right)}\left(t\right),\cdots ,R_{n}^{\left(M\right)}\left(t\right)$ bit/s, as long as $\sum_{m=1}^{M}R_{n}^{\left(m\right)}\left(t\right)=R_{n}\left(t\right)$. With this assumption, we can ignore some unconcerned physical layer details and focus on the topological aspects of the network that determine the capacity. Thus, our results can be readily extended to incorporate the situation where each link has a different and known capacity.

## 4. Network Capacity

In this section, we develop a mathematical model that assesses the network capacity of an arbitrary sensor and satellite network as mentioned above. Denote the above network by $G\left(V_{N},E\right)$. The network capacity is the total amount of data that can be exchanged across a network over a finite time.

Let $v_{i}\in V_{N}$ be a source node, and let $b_{i,j}$ be the *j*-th bit transmitted from $v_{i}$ to its destination $d\left(v_{i},j\right)$. In unicast scenarios, $d\left(v_{i},j\right)$ represents a single destination; and for multicast, $d\left(v_{i},j\right)$ represents the set of all destinations. Let $A_{i,T}^{\chi }$ be the amount of bits transmitted by $v_{i}$ and which successfully reached their respective destinations during the time interval $\left[t_{0},t_{0}+T\right]$, where $t_{0}$ is the start time. We take $A_{i,T}^{\chi }$ as the capacity of source node $v_{i}$. χ∈X in $A_{i,T}^{\chi }$ denotes the spatial and temporal network scheduling algorithm, and X denotes the set of all scheduling algorithms. If the same bit is transmitted from a source $v_{i}$ to multiple destinations $D\left(v_{i},j\right)$, *i.e*., multicast. All of the bits are the same, so only one bit is counted in the calculation of $A_{i,T}^{\chi }$. Then, we assume that the network is stable ∀χ∈X. A network is stable if and only if for any fixed *N*, each node in the network has an infinite queue to transmit, and the queue length in any relay node storing packets in transit does not grow to infinity as T→∞. That is, with an infinite queue to transmit, the long-term incoming data rate into the network equals the outgoing data rate. We further assume that there is no traffic loss for queue overflow.

The transport capacity of an arbitrary sensor and satellite network $G\left(V_{N},E\right)$ when using the spatial and temporal network scheduling algorithm *χ*, denoted by $C_{G}^{\chi }$, is defined as:

(1) $$C_{G}^{\chi }\overset{\Delta }{\;=}\frac{\sum_{i=1}^{N}A_{i,T}^{\chi }}{T}$$

Additionally, the maximum transport capacity of the network is defined as:

(2) $$C_{G}\overset{\Delta }{\;=}\max_{\chi \in X}C_{G}^{\chi }$$

Obviously, for any *χ*, we have $C_{G}\geq C_{G}^{\chi }$.

The amount of bits successfully transmitted by the source (the capacity of) is defined as:

(3) $$\begin{array}{c} A_{i,T}^{\chi }=\sum_{d\in D_{i}}\int_{t=t_{0}}^{T+t_{0}}\left(\sum_{p\in P_{i}^{d}}\phi_{i}\left(d,p,t\right)r_{i}\left(d,p,t\right)s_{i}\left(d,p,t\right)\eta_{i}\left(d,p,t\right)\right)dt \end{array}$$

In Equation (3), $D_{i}$ is the set of all destination nodes of $v_{i}$ in $\left[t_{0},t_{0}+T\right]$, and $P_{i}^{d}$ is the set of all paths from $v_{i}$ to the corresponding destination node *d*. $\varphi_{i}\left(d,p,t\right)$ repents the availability of the path *p* (existence of a line-of-sight between nodes in the path) from the source node $v_{i}$ to the destination node *d* at time *t*. The dynamic data transfer rate is denoted by $r_{i}\left(d,p,t\right)$ and is characteristic of the IWSSN systems. The establishment of the path *p* from $v_{i}$ to *d* is driven by the spatial and temporal network scheduling algorithm *χ* and is denoted by $s_{i}\left(d,p,t\right)$. $\eta_{i}\left(d,p,t\right)$ is the efficiency function of path *p* scheduled by *χ*. The total amount of bits successfully transmitted by source $v_{i}$ is computed by summing the integrated data transfer rates to each destination node over the full time period $t\in \left[t_{0},t_{0}+T\right]$. The four components of $A_{i,T}^{\chi }$ can be used to populate the following matrices to aid in implementation: $\varphi_{i}\left(d,p,t\right)\in \Phi_{i}{\left(t\right)}^{M\times Q}$, $r_{i}\left(d,p,t\right)\in R_{i}{\left(t\right)}^{M\times Q}$, $s_{i}\left(d,p,t\right)\in S_{i}{\left(t\right)}^{M\times Q}$ and $\eta_{i}\left(d,p,t\right)\in H_{i}{\left(t\right)}^{M\times Q}$, where $M=\left|D_{i}\right|$, $Q=\;\max_{d\in D_{i}}\left(\left|P_{i}^{d}\right|\right)$, and $\left|\cdot \right|$ denotes the number of elements in the set.

(1) Availability: The first component of the source node capacity model is based on the existence of path *p*. Additionally, this is dependent on the line-of-sight of each link in the path *p* as a function of the orbital dynamics of the satellites, the position of sensor nodes, the minimum elevation visibility constraints and time. The availability matrix is denoted by $\Phi_{i}{\left(t\right)}^{M\times Q}$, consisting of elements $\varphi_{i}\left(d,p,t\right)\in \left\{0,1\right\}$, $\forall d\in D_{i}$, $p\in P_{i}^{d}$, $t\in \left[t_{0},t_{0}+T\right]$. Where an available path *p* between the source node $v_{i}$ and the destination node *d* is expressed as $\varphi_{idp}\left(t\right)=1$ and when the path is not available or does not exist, the corresponding element in the matrix is assigned to zero.

(2) Transfer rate: The data transfer rate matrix is denoted by $R_{i}{\left(t\right)}^{M\times Q}$, where the data transfer rate between source node $v_{i}$ and destination node *d* in the path *p* is $r_{idp}\left(t\right)$, $d\in D_{i}$, $p\in P_{i}^{d}$, $t\in \left[t_{0},t_{0}+T\right]$. Generally, the data transfer rates are selected based on expected channel performance (*i.e.*, constrained by the minimum signal-to-noise (SNR) requirements) and can be updated during the operation of the network [40,41]. The optimal link rate distributions may be selected by network scheduling algorithm *χ* to maximize the throughput.

(3) Establishment of the path: Governed by the network scheduling algorithm χ∈X, a path *p* may or may not be wanted even if it is available. The establishment matrix is denoted by $S_{i}{\left(t\right)}^{M\times Q}$, and the wanted path *p* between source node $v_{i}$ and destination node *d* is denoted by $s_{idp}\left(t\right)=1$, where $d\in D_{i}$, $p\in P_{i}^{d}$, $t\in \left[t_{0},t_{0}+T\right]$. If network scheduling algorithm *χ* does not allow *p* to transfer data, $s_{idp}\left(t\right)=0$.

(4) Path efficiency: Successful data transfer from source node $v_{i}$ to destination node *d* is influenced by all of the nodes in the path *p*. Nodes in the path may not maintain perfect links due to some reasons, e.g., antenna slewing and acquisition maneuvers, unknown noise that degrades the SNR and system failures. A node $v_{r}\in p$ that always operates perfectly has an efficiency factor $\eta_{r}\left(t\right)=1$, $\forall t\in \left[t_{0},t_{0}+T\right]$. While a node $v_{r}\in p$ is available on average 95% of the time, $\eta_{r}\left(t\right)=0.95$, $\forall t\in \left[t_{0},t_{0}+T\right]$. The path efficiency is defined as $\eta_{i}\left(d,p,t\right)\overset{\Delta }{\;=}\prod_{v_{r}\in p}\eta_{r}\left(t\right)$. The path efficiency matrix is denoted by $H_{i}{\left(t\right)}^{M\times Q}$, and the efficiency of path *p* between $v_{i}$ and *d* is denoted by $\eta_{idp}\left(t\right)$.

## 5. Capacity Trends Analysis

In this section, we utilize the capacity model proposed earlier to assess the network capacity trends of representative sensor and satellite networks. First, the simulation tools and the environment are described. Second, we analyze the major constraints in the network capacity model, including the dynamics of nodes, long-term variations, the number of nodes and actualized factors. Third, we study the relationships between the capacity model and major constraints. From the analysis, we obtain the network capacity trends and find the opportunities to optimize the IWSSN network schedules.

### 5.1. Simulation Environment Description

We combine the geometric satellite orbit model and the satellite tool kit (STK) engineering software to quantify the trends of the capacity constraints. Simulation results in this section were carried out jointly using STK and MATLAB. Firstly, satellite orbital propagators and remote sensor locations of the initial epoch in a MATLAB script are loaded into STK through the interface between STK and MATLAB. Then, STK is used to calculate node parameters (e.g. position, velocity, *etc*.) and link parameters (e.g., range, duration, azimuth, elevation, *etc*.). Finally, node and link parameters generated from STK are exported to MATLAB for further processing and analysis.

Our simulations extract node settings from a variety of sources to reflect realistic satellite and sensor networks. Satellite data settings are generated referring to the union of concerned scientists (UCS) satellite database [42] from http://www.ucsusa.org/. Information on remote sensors is drawn from the default datasets in STK.

### 5.2. Dynamics of Nodes

We now study the impact of dynamic node positions on availability in the network capacity model. The IWSSN is a high dynamic network. Satellites are located in orbits above the Earth and are usually mobile relative to the ground. New satellites and sensors may inject into the IWSSN, and invalid nodes may also be ruled out from the network. Although satellites in different orbits may be mobile relative to each other, in most practical scenarios, inter-satellite links (ISLs) are designed to be stable (ISLs are generally designed to connect satellites in the same orbital plane or satellites in adjacent orbital planes with slow relative movement in most practical space scenarios, e.g., ISLs in Iridium [38,39], advanced extremely high frequency (AEHF) [43] and tracking and data relay satellite (TDRS) [44] systems; there may be high dynamic ISLs, such as in multi-layered satellite network (MLSN) [45]; the special cases are not considered in our current work). Hence, the main factors affecting the availability are the line-of-sights between satellite nodes and sensor nodes.

In order to study the impact of dynamic node positions on the network capacity model. We focus on the interaction of nodes’ dynamics and availability in Equation (3), *i.e*., we study capacity uniquely as a function of availability time. Hence, we only consider $\varphi \left(d,p,t\right)$ and assume that the other constraints $r_{i}\left(d,p,t\right)$, $s_{i}\left(d,p,t\right)$ and $\eta_{i}\left(d,p,t\right)$ in Equation (3) are constant. Then, from Equations (1) and (3), capacity is directly proportional to availability time and can be expressed as:

(4) $$C_{G}^{\chi }\propto \frac{1}{T}\sum_{i=1}^{N}\sum_{d\in D_{i}}\sum_{p\in P_{i}^{d}}\int_{t_{0}}^{T+t_{0}}\varphi_{idp}\left(t\right)dt$$

where $\varphi_{idp}\left(t\right)$ is equal to $\varphi_{i}\left(d,p,t\right)$. We are interested in the major contributors to the path availability $\varphi_{idp}\left(t\right)$, *i.e*., the dynamically-varying duration of each satellite node in the orbit pass over visible remote sensor nodes. Hence, we should determine if there is visibility between satellite nodes and sensor nodes; that is, if the sensor node is in the coverage area of a satellite during the orbit.

**Figure 2:**
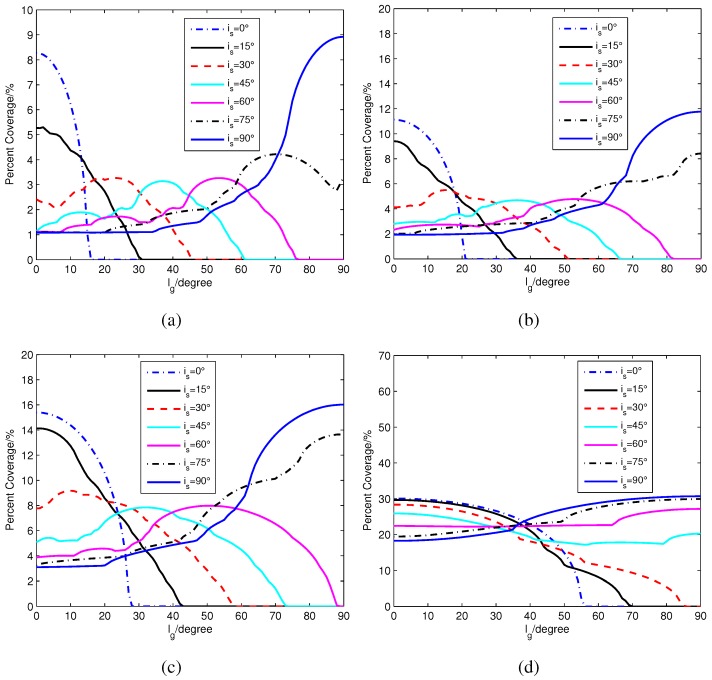
Percentage of satellite passes that can cover the sensors for different satellite inclinations and sensor node latitudes with $e_{\min}=10^{\circ }$. (**a**) Satellite altitude: 550 km; (**b**) Satellite altitude: 880 km; (**c**) Satellite altitude: 1450 km; (**d**) Satellite altitude: 8050 km.

The percentage of passes that results in visibility between a satellite node and remote sensor node can be evaluated using simple geometric model as a function of satellite inclination, altitude and the sensor latitude [46]. We verify this model in our simulation, as shown in Figure 2. The altitudes of the satellites are 550 km, 880 km, 1450 km and 8050 km, respectively. We select these altitudes from low to high, and the recursive orbit period of these altitudes are all two days. Statistical coverage analysis is done for satellite inclinations $i_{s}$ and the sensor latitudes $l_{g}$ in the Northern Hemisphere (*i.e*., $0^{\circ }\leq i_{s}\leq 90^{\circ }$ and $0^{\circ }\leq l_{g}\leq 90^{\circ }$). The minimum elevation angle $e_{\min}$ at which the remote sensor can establish a link to an orbiting satellite in the simulation is $10^{\circ }$. From Figure 2, the general trend is that visibility duration increases as the satellite inclination approaches the sensor’s latitude. Obviously, satellites with low inclinations only cover equatorial and near-equatorial areas, while satellites with polar orbits cover remote sensors with all latitudes. The percent coverage of low latitude areas is reduced compared to high latitude areas when satellite inclination increases. Useful information about the visibility duration trends can be extracted from Figure 2 as a function of satellite and sensor node parameters. For instance, under the network topology constraint, a satellite with inclination $i_{s}=30^{\circ }$ cannot cover any sensor nodes with latitudes $l_{g}>51^{\circ }$ for an 880-km orbit and $l_{g}>58^{\circ }$ for a 1450 km orbit. For satellites with low inclinations, the percent coverage curves in Figure 2 generally move towards higher latitudes when the satellite altitude increases. The range of latitudes with zero coverage decreases about $15^{\circ }$ with the increase in inclination.

To compute total visibility duration, we first calculate the satellite’s position vector $W\left(t\right)$ in the Earth-centered Earth-fixed (ECEF) frame with the initial satellite orbit parameters (semimajor axis, eccentricity, inclination, argument perigee, right ascension of the ascending node (RAAN) and true anomaly) using the geometric algorithm introduced in [47], where $t\in \left[t_{0},t_{0}+T\right]$. The position vector of the satellite in the ECEF frame is time-varying, but $W\left(t\right)$ in each moment can be easily calculated based on the initial orbit parameters. Then, we calculate the position vector *U* of the sensor node in the ECEF frame with the longitude and latitude parameters. Obviously, the position vector of the sensor node in the ECEF frame is static. Hence, the visibility between the satellite and the sensor node can be expressed as:

(5) $$\psi \left(t\right)=e_{\min}-\arccos\left(\frac{W\left(t\right)\cdot U}{\left|W\left(t\right)\right|\left|U\right|}\right)$$

If $\psi \left(t\right)\geq 0$, there is visibility between the satellite node and the sensor node. Additionally, the visibility duration ΔT can be expressed as:

(6) $$\Delta T=\int_{t_{0}}^{T+t_{0}}\varepsilon \left(\psi \left(t\right)\right)dt$$

In Equation (6), $\varepsilon \left(t\right)$ is the unit step function, where, if t≥0, $\varepsilon \left(t\right)=1$, otherwise $\varepsilon \left(t\right)=0$.

From the visibility duration trends in this section, optimal satellite orbits and sensor node locations for maximizing path availability, *i.e*., capacity, can be selected using the equations and plots presented.

### 5.3. Long-Term Variations

**Figure 3:**
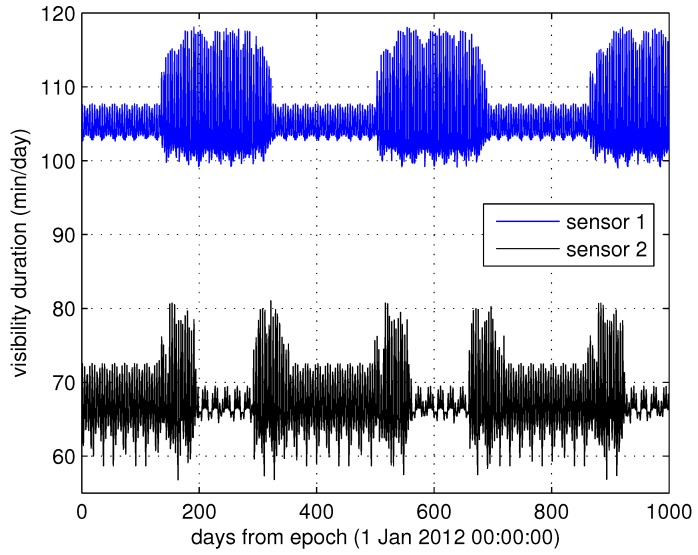
Long-term visibility duration variations between the satellite and two ground stations with $e_{\min}=0^{\circ }$.

Variations in satellite trajectory can be categorized into two classes: (1) short-term (one or several orbital cycle) variations as described in Section 4.2; and (2) long-term (or seasonal) variations. In high-risk natural disaster areas (e.g., seismic zones), sensors are usually distributed in the long term [1]. The long-term satellite trajectory variations usually have distinct periods and so does the visibility duration dominated by the trajectory. Figure 3 is the simulation result of long-period oscillation of visibility duration variations. In this simulation, satellite orbit parameters are in $J_{4}$ ( body’s fourth dynamic form factor) gravity coefficient [48], as shown in Table 1. Two ground stations are Mohe at ($53.4^{\circ }$ N, $122.3^{\circ }$ E), and Sanya at ($18.2^{\circ }$ N, $109.5^{\circ }$ E) respectively.

This oscillation is a very significant trend for network capacity. Space missions are generally designed for the worst case scenario to guarantee their mission objectives will be satisfied and sacrificing the potential advantages in better cases. From the simulation result in Figure 3, it is obvious that if we deal with these naturally-occurring long-term variations well, the network capacity can be improved.

**Table 1:** Satellite orbit parameters.

Parameters	Value
Propagator	$J_{4}$ Perturbation
Start Epoch	1 January 2012 00:00:00.00 UTCG
Stop Epoch	1 January 2015 00:00:00.00 UTCG
Orbit Epoch	1 January 2012 00:00:00.00 UTCG
Apogee Altitude	800 km
Perigee Altitude	800 km
Inclination	60${}^{\circ }$
Argument of Perigee	0${}^{\circ }$
RAAN	0${}^{\circ }$
True Anomaly	0${}^{\circ }$

The long-term visibility duration variations can be largely explained by the perturbations of satellite rotational rates due to Earth’s oblateness characterized by the $J_{4}$ gravity coefficient [48]. The length of each visibility duration is directly related to the maximum elevation angle $e_{\max}$ between the remote sensor and the satellite, where $e_{\max}$ can be calculated using:

(7) $$e_{\max}=\frac{\pi }{2}-\theta -\text{arcsin}\left(\frac{r_{E}\sin\theta }{\sqrt{\left(r_{E}^{2}+r^{2}-2r_{E}r\cos\theta \right)}}\right)$$

In Equation (7), *θ* is the Earth central angle measured at the center of the Earth from the sub-satellite point to the remote sensor when the longitudes of the sub-satellite point and the sensor are equal. Additionally, the sub-satellite point is the intersection point of a straight line from the satellite to the center of the Earth and the Earth’s surface. The maximum elevation angle $e_{\max}$ dominates the visibility duration of each pass and varies with the successive passes. It is obvious from Equation (7) that the remote sensor always lies in the orbit plane of a satellite that has very little variation in visibility duration (*θ* will be zero, and emax=ππ22); for example, an equatorial remote sensor and a satellite with zero inclination. Since those satellites orbits and sensors are not in the same plane, we are interested in the amplitude and frequency of visibility duration variability over successive passes. As many of the trends are periodic in nature, models to characterize the variations can be developed. With these models, space missions need not to be designed for the worst case. Additionally, potential advantages in better cases can be utilized for maximizing network capacity.

### 5.4. Number of Nodes

It is obvious that larger networks (with more nodes and links) provide greater opportunities for data transmission. However, the network capacity does not scale linearly with the size of the network. For example, if there are multiple sensors communicating with each single satellite, satellite *m* can communicate only with $s_{m}$ sensors at a given time instant. In this simulation, each satellite can only support 20 sensors with $e_{\min}=10^{\circ }$. Seventy satellites are launched into 500 to 1500 km altitude random orbits. Additionally, the visibility duration is calculated relative to the increasing number of sensors that is randomly distributed in the area between 40${}^{\circ }$ S and 40${}^{\circ }$ N on the Earth. The following figures are the results with 1000 Monte Carlo simulations.

**Figure 4:**
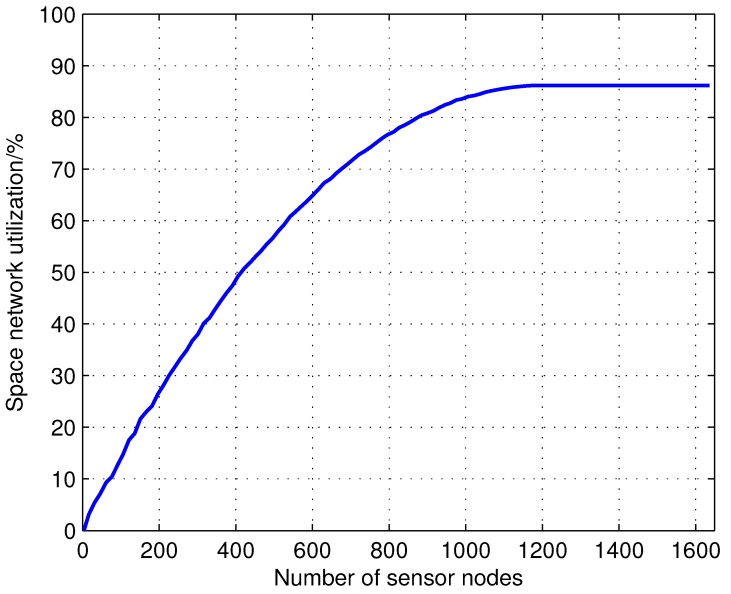
Effects of a growing set of sensor nodes on space network utilization.

Figure 4 shows the simulation results when the number of sensor nodes that are injected into the IWSSN increase; the utilization of the satellite increases until a saturation point is reached, where additional sensor nodes can no longer be supported. As increasing numbers of sensor nodes are injected into the network, the saturation point of the satellite network in the sensors distribution area will be reached. It is 87.21% of the whole space network resource according to the statistical results, and the other 12.79% space network resource cannot cover the sensor distribution area dominated by the satellite trajectories. When the saturation point is reached, additional sensor nodes can no longer be supported, and the capacity of the network will not increase. The network capacity when considering the establishment of the path will be less than or equal to the summed total visibility durations.

**Figure 5:**
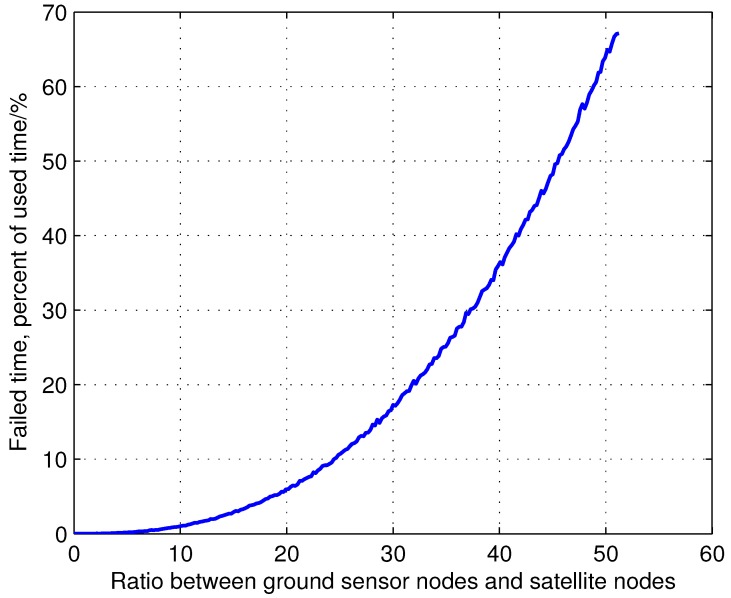
The effects of the growing ratio between the ground sensor nodes and satellite nodes on failed access time for the sensor nodes. The failed access time is the time when sensors cannot access satellites due to the capacity constraints of the satellite network. The scenario of this result is identical to the one of Figure 4. When the ratio between ground sensor nodes and satellite nodes is 50, there are 3500 sensor nodes in the network.

Figure 5 shows failed access time, defined as the time when sensors cannot access satellites due to the capacity constraints of the satellite network. The growth of failed access time is exponential with the growing ratio between ground sensor nodes and satellite nodes, which is nearly or already at capacity, rapidly reducing the overall satellite network utilization. Hence, from Figure 4 and Figure 5, it is obvious that realistic scheduling constraints must be considered in network scheduling algorithm χ∈X. Intelligent node deployment methods and network scheduling algorithms are critical to maximize the network capacity of the growing IWSSN.

### 5.5. Actualized Factors

Recall that nodes in the network may not maintain perfect links due to some actualized factors, e.g., antenna slewing and acquisition maneuvers, unknown noise that degrades the SNR and system failures. In high fidelity network capacity models, these inefficiencies and constraints of the network should be considered, both from the satellite-satellite and sensor-satellite perspectives.

**Figure 6:**
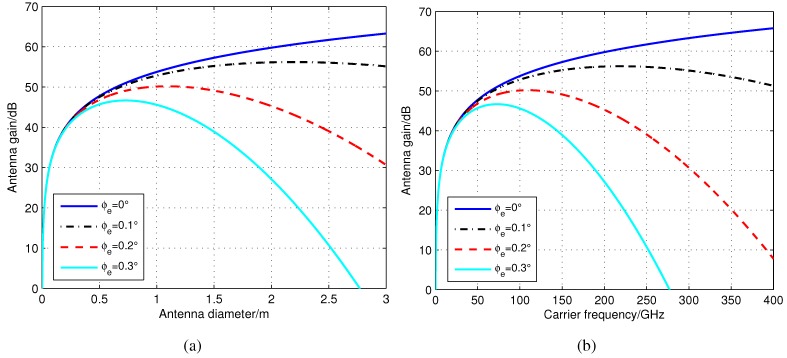
Gain of the inter-satellite links (ISLs) antenna of different alignment error angles. (**a**) Variation with antenna diameter (the carrier frequency is 60 GHz, and the efficiency of the antenna is 0.6); (**b**) Variation with carrier frequency (the antenna diameter is 0.6 m, and the efficiency of the antenna is 0.6).

A path *p* from the source node $v_{i}$ to the destination node *d* may contain ISLs and sensor-satellite links (SSLs). ISLs are usually based on high frequency band (Ka, EHF or laser), and their beam-widths are narrow. In addition, the attitude control of each satellite usually has certain errors. All of these make the transmitting and receiving antennas off the trail, *i.e*., alignment errors. Alignment errors also exist in SSLs, but the beam-widths in SSLs are broader, so the performance deteriorations are not as obvious as that in ISLs. Due to the alignment errors of the transmitting and receiving antennas, the actual antenna gain is lower than the antenna peak gain. The decrease of the gain, *i.e*., alignment loss, can be approximately calculated using Loss=11.1ϕeϕeϕ3dBϕ3dBdB from [49], where $\phi_{e}$ is the alignment error angle, ϕ3dB≈70λ70λDD is the 3-dB beam-width of the antenna. In $\phi_{3\mathrm{dB}}$, $\lambda \left(m\right)$ denotes the carrier wave length and $D\left(m\right)$ denotes the antenna diameter. Figure 6 shows the antenna gain when alignment error angle $\phi_{e}$ varies from $0^{\circ }$ to $0.2^{\circ }$. As a result, if $\phi_{e}=0^{\circ }$, antenna gain increases with the rise of antenna diameter and carrier frequency. When $\phi_{e}\neq 0^{\circ }$, antenna gain increases until the highest point and then decreases. Alignment loss makes node $\forall v_{r}\in p$ not be able to operate perfectly, *i.e*., $\eta_{r}\left(t\right)<1$, $\eta_{i}\left(d,p,t\right)=\prod_{v_{r}\in p}\eta_{r}\left(t\right)<1$. Additionally, from Figure 6, the optimal antenna diameter and carrier frequency of the space nodes can be selected for a maximum network capacity before they are injected into the IWSSN.

When focusing on the sensor-satellite perspective, more actualized factors should be considered. The actual time that a link can be maintained is usually less than the total visibility duration. Let $\beta_{t}$ be the actual time the data link is maintained relative to the total visibility durations between the sensor and satellite. $\beta_{t}$ is usually less than 100% and is tightly related to the actualized factors, such as maximum elevation angle $e_{\max}$, carrier frequency $f_{c}$, average rainfall rate $R_{rain}$, altitude of the sensor node $h_{s}$, *etc*. What is more, considering the above actualized factors and based on the minimum SNR requirement, the maximum transfer rate of the SSLs may also vary when using adaptive coding and modulation (ACM) [50,51].

Obviously, higher fidelity satellite and sensor models will further decrease the network capacity and limit the optimal solution space of the network scheduling. Optimal network scheduling should not only consider paths with fewer hops, but also consider many actualized factors, even including the local weather.

### 5.6. Relationships Between the Capacity Model and Major Constraints

**Figure 7:**
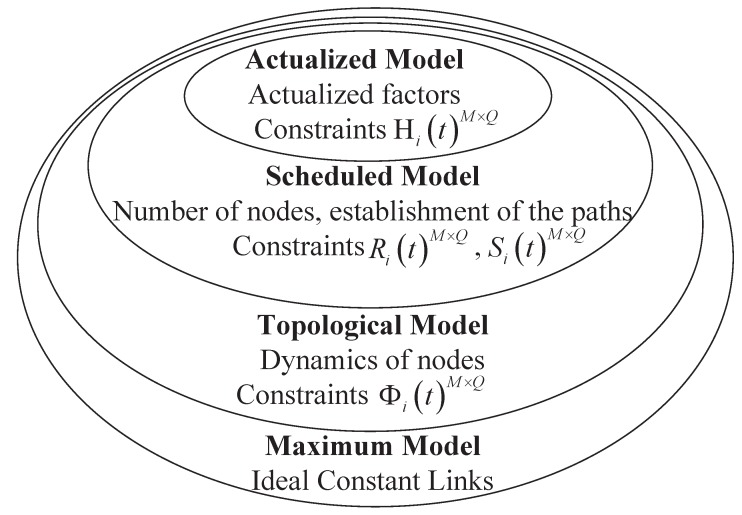
Relationships between the network capacity model and major constraints. This is a schematic diagram with an increasingly higher fidelity network capacity model within smaller ellipses, where the ellipse area represents network capacity.

In order to conclude the relationships between the network capacity model and major constraints, we now introduce a four-layered network capacity model as shown in Figure 7. With every additional model layer, different classes of constraints are progressively considered to get a more realistic representation. That is, each layer describes successively higher model fidelity, and network capacity generally results in a reduction.

The maximum model assumes ideal constant availability between communication nodes, such that the elements of $\Phi_{i}{\left(t\right)}^{M\times Q}$ are one. The first level is used to characterize the overall maximum throughput rate. Then, we assume the link availability as a function of the nodes’ dynamics. This layer falls into the framework of the topological model. Network scheduling constraints are introduced in the higher fidelity scheduled model. The number of nodes and conflicting satellite schedules are key parameters in the scheduled level. Link efficiency is considered in the final actualized model and includes many actualized parameters.

## 6. Conclusions and Future Work

We investigated the capacity problem of IWSSN. The motivation was to provide insights and design guidelines for optimizing IWSSN in emergency scenarios. Firstly, we formulated a general model to evaluate the remote sensor and satellite network capacity. Four major constraints were introduced, including availability, transfer rate, establishment of the path and path efficiency. Then, we combined the geometric satellite orbit model and STK engineering software to quantify the trends of the capacity constraints in the simulation section. We discussed each major capacity constraint, exemplify their impacts with representative networks and showed the opportunities to optimize the IWSSN network schedules through intelligent deployment and flexible scheduling.

However, there are important issues that are still open and should be further investigated in the future. Specifically, although the arbitrary sensor and satellite network capacity model is provide with major capacity-impacting constraints, it is noted that more high fidelity constraints can be considered, such as energy limits, satellite attitude control and data processing. It is also seen that we only show the optimizing opportunities of IWSSN network schedules in this paper. Optimal scheduling algorithms that seek to maximize a capacity-related objective function will be developed in our future work. Furthermore, we are developing models and algorithms to calculate the exact network capacity value for IWSSN based on our current network capacity model.
